# The Chicago Gynecological Society, Meeting of July 17th

**Published:** 1885-10

**Authors:** 

**Affiliations:** 233 Indiana avenue


### SOCIETY REPORTS.

---

TRANSACTIONS OF THE CHICAGO GYNECOLOGICAL SOCIETY.

*Regular meeting, 17th July,* 1885.

I.—NEWMAN. Some Considerations Relating to Shock and Nervous Influence in Parturition.

II.—FOSTER. On the Diet and Management of Artificially Fed Infants.

DR. W. H. BYFORD in the chair.

I. An inaugural thesis, entitled, "SOME CONSIDERATIONS RELATING TO SHOCK AND NERVOUS INFLUENCE IN PARTURITION," by HENRY P. NEWMAN, M. D., (Detroit Medical College, 1878), was read by the Secretary, DR. EDWARD WARREN SAWYER. DR. NEWMAN related the histories of three cases of nerve-exhaustion and shock, occurring during parturition. In conclusion, he desired to offer the following deductions:

"1st. That we have a higher nervous organization presiding over the process of childbirth and subjecting it to like influences and derangements which obtain in other physiological functions.

"2nd. As civilization advances, the co-relation of mind and matter becomes more intimate and complex, and calls for a proportionate advance in psychological therapeutics, and the application thereof to cases of predominant mental and nervous influences.

"3rd. In many cases of so-called tedious labours the irregular contractions of the first stage are the result of an exalted state of nervous irritability.

"4th. Active interference is indicated in many cases of protracted labour due to nervous influence, to guard against the dangers of exhaustion and shock.

"5th. Much is to be expected from judicious prophylaxis. Especially would I urge the necessity of direct professional supervision over the entire period of gestation from the earliest months.

"6th. There will still remain to be combated social, moral and educational environments, which we can scarcely expect to see abolished, until the laity, as well as the profession, is better informed as to the deleterious consequences of departure from the standard of physiological perfection in the mothers of our race, and the best means of approximating that equipoise of the mental and physical organization which it is primarily the design of nature to establish."

The paper is published *in extenso* in the CHICAGO MEDICAL JOURNAL AND EXAMINER, August, 1885.

DISCUSSION.

DR. HENRY T. BYFORD thought DR. NEWMAN'S essay was of great practical interest. In every nation, savage or civilized, injurious customs gradually grow up in the methods of managing labour, and require a periodical weeding out. They affect both the profession and the laity, and are the principal causes of the troubles referred to, and should receive attention.

II. An inaugural thesis, entitled "THE DIET AND MANAGEMENT OF ARTIFICIALLY-FED INFANTS," by ADDISON H. FOSTER, M. D., (College of Physicians and Surgeons of New York, 1866), was read by the Secretary. DR. FOSTER'S conclusions were:

"1st. The artificially fed city infant too often inherits an enfeebled nervous and physical organization.

"2nd. Its surrounding are rarely of an invigorating character.

"3rd. It usually has a desperate struggle for life with an imperfect diet, for even new milk from the prairie-fed Chicago cow is likely to be harmful.

"4th. The wonder is, that so many escape death at the hands of over-anxious but unwise attendants."

Dr. Foster's paper is published *in extenso* in the CHICAGO MEDICAL JOUFNAL AND EXAMINER, August, 1885.

DISCUSSION.

DR. E. J. DOERING said, that as the majority of his patients were children, he had given the subject of infant-feeding a great deal of attention. He favored, of course, a wet-nurse, whenever the mother could not nurse, and always urged a removal into the country during the summer. If this was impracticable, he preferred condensed milk for the first three months. After that age, until the age of six months, he prefers a mixture of varying proportions, according to age, of barley-water and fresh cow's milk, to which a little sugar ot milk is added. The addition of barley-water causes a mechanical separation of the particles of curd, and facilitates digestion. After the age of six months he allows farinaceous food to be added, preferring the preparation known as Imperial Granum. Mellin's and Horlick's foods are also valuable, particularly if there is a tendency to constipation. These two latter preparations may be given to young infants, if their digestive power is good. If there is much gastric derangement, he prefers peptonized milk, and believes that in the absence of mother's milk, cow's milk, properly prepared for the infant, answers every requirement.

PROFESSOR CHARLES WARRINGTON EARLE said, that the subject of infant-feeding was one of the most important which occurred to him in practice. He urged every woman to nurse her own baby, but if this was impossible, he suggested a wet-nurse.

Sometimes the circumstances of the family would not permit a wet-nurse, and the mother may furnish a portion of its nutriment, and the child comes up on a mixed diet—part mother's milk and part artificial food But every year, more and more wet-nurses are employed, and infant mortality is lessened. If artificial food is used, there should be enough of it. Many children lose their lives every year from starvation, and this is particularly true if condensed milk is used; not that the condensed milk is not a good diet in some cases, but it is only condensed four or five times, and a sufficient quantity is not used to nourish the child. One teaspoonful to eight of some diluent will agree often with a very young babe, and in older children he gives from one-half to one can of condensed milk daily, diluted with barley or rice-water. Professor Earle's favorite food for a young babes, deprived of its natural diet, was cream and barley-water slightly sweetened and salted. Murdock's food, in doses of from one to ten drops, had been of value.

DR. H. T. BYFORD said: There is but one proper food, or basis of food, for infants under six months, and that is the mother's milk. There never has been a proper food for them that has not been prepared mainly from the milk of some animal.

Farinaceous articles, even when largely converted into glucose or grape sugar, as in the Liebig foods, cannot be digested in sufficient quantities to sustain vigorous life at this age. Whether mixed with diluted milk or not, they either induce acid indigestion, or else pass through the alimentary canal in sufficient quantity to absorb the natural secretions, and appear at stool as hard, irritating masses. He has seen Ridge's food, which is so well adapted for older children, produce fatal entero-colitis when given during the first five or six months of life; and has seldom been able, in warm weather, to give Mellin's food, one of the best of the Leibig class, in more than quarter or half the amount necessary for nutrition, without sooner or later injuring their digestive systems. Indigestion, or poor nutrition, or both, are common proofs of the unfitness of the so-called milk foods, which contain cane and grape sugars and starch in large quantities.

Condensed milk, after proper dilution, is but cow's milk deprived of about one-half of its fat, the most important ingredient for these very young children. It often agrees well with the stomach, but, unless other food is given with it, produces very poorly-nourished children. Peptonized milk is better than ordinary cow's milk, chiefly because curdling and fermentative changes are prevented, and it does not call for much less solvent and absorbent power in the stomach, nor for much less bile in the duodenum than other milk. It is a degree better than milk that has been scalded while fresh, and several degrees worse than milk warm from the udder. The reason why goat's milk is often so well borne, is because it is drank as soon as milked.

Fat is, perhaps, the only artificial food that closely resembles anything found in milk, and as fat is easily digested almost from the time of birth, and is by far the most concerned in infant nutrition, it must be the chief constituent of their diet. Hence, while simple dilution may be the best method of treating milk warm from the cow, after it has stood awhile the upper third or quarter should be used, diluted with two or three parts of lime, or cracker, or barley-water, and slightly sweetened with sugar of milk. As an adjuvant, where none but a very dilute diet is borne, he has seen Trommer's extract of malt with cod liver oil, in teaspoonful doses, or a little good bacon, taken with benefit.

On motion, DRS. HENRY P. NEWMAN and ADDISON H. FOSTER were elected Fellows of the Society.

W. W. JAGGARD, M. D., *Editor.*

233 Indiana avenue.
September, 1885.

